# Is gamification a good approach to influence pharmacists’ behaviour?

**DOI:** 10.1177/17151635221074956

**Published:** 2022-02-01

**Authors:** Rand Hussein, Kelly A. Grindrod

## Introduction

The pharmacist’s role continues to evolve and expand from the traditional role of dispensing toward a more patient-centred model of care. However, many pharmacists still fall short in terms of their provision of full-scope pharmacy services.^1-4^ Behaviour change interventions such as educational meetings and printed educational materials have had little effect on the behaviour of health care professionals (HCPs), including pharmacists.^5,6^ Thus, a new behaviour change intervention is needed to influence pharmacists’ behaviours. One such intervention is gamification, which is defined as the use of game elements in nongame contexts.^7^ Gamification is different from serious games, which are fully immersive games designed for education and training.^8^ Gamification uses a combination of game elements such as points and badges to create gamelike experiences that enhance users’ motivation and engagement with desired behaviours.^9,10^

## Gamification use in continuing professional development for HPCs

Gamification has been heavily applied in the domains of education, training, health and marketing^11,12^ and has generally enhanced engagement with desired behaviours and provoking behaviour change.^12-14^ The use of gamification has been well established to achieve general wellness by increasing physical activity and healthy eating habits.^12,13,15^ It can also improve the way patients cope with their chronic medical conditions, especially in children and adolescents with diabetes.^16,17^ However, when it comes to HCPs, most of the literature focuses on the value of gamification in educating younger users such as medical students and residents. Studies have shown promising results in improving knowledge retention,^18^ critical thinking and confidence in applying knowledge gained from the classroom to real-life experiences.^19^

The use of gamification to influence HCPs’ behaviour is less well explored. Of the few examples identified, most focused on the influence of gamification on the behaviour of physicians and nurses. For example, the “150 lives in 150 days” campaign by McKeown et al.^20^ used a gamified approach combining points, leaderboards, time pressure, stories and badges to address emergency department physicians’ low adherence to a sepsis protocol in British Columbia. The results showed that the campaign significantly improved physicians’ compliance with and awareness of the sepsis protocol, which was sustained for up to a year after the campaign was over. In addition, the mortality rate of patients with severe sepsis dropped from 21% to 6%.^20^ The “Helping Hippocrates” project by Greenly also used the concept of gamification, combining points, badges and feedback to enhance health care worker compliance with checking patients’ wristbands. The project reduced patient identification errors from 8.2% to 0%.^21^

## Explaining how gamification can influence pharmacists’ behaviour

Gamification can influence pharmacists’ behaviour by motivating them to engage in desired activities. There are 2 types of motivation that need to be considered when designing a gamified intervention: extrinsic and intrinsic motivation. Extrinsic motivation refers to the tendency to engage in an activity to either attain a reward or to avoid a punishment.^22^ In contrast, intrinsic motivation refers to the tendency to engage in an activity for the benefits of doing the activity itself (i.e., because it is satisfying or enjoyable).^23^ Critically, extrinsic motivation can be used to help someone develop intrinsic motivation, but extrinsic motivation can also undermine intrinsic motivation if users engage in an activity only for the rewards.^24^ Thus, a well-designed gamified intervention may use an external motivator such as points or badges to get users to start a behaviour, particularly one that is perceived as boring or uninteresting, but that may drop off over time.^24,25^ Further, rewards can be used selectively to encourage the repetition of a new skill or behaviour and, again, drop off as the user masters the skill and appreciates its value in real life.^24,26^ For example, pharmacists can be extrinsically motivated by receiving a gift card as a reward for practising a new behaviour (e.g., administering vaccines for the first time, dispensing naloxone kits), and once they master the skills through repetition, the gift card will no longer be necessary to drive behaviour. Ultimately, interventions that target extrinsic motivation will tend to produce short-term change, but the goal is to improve intrinsic motivation to achieve a sustained change in behaviour.^20^

While extrinsic motivation is generally addressed through rewards, intrinsic is more complex. One reason to focus on intrinsic motivation is that it is more aligned with performance and creativity.^23,25^ Self-determination theory has been widely used to explain how gamification influences intrinsic motivation to affect behaviours.^27-29^ The theory posits that intrinsic motivation is targeted by satisfying 3 psychological needs: autonomy, competence and social relatedness.^28,30-32^ Autonomy refers to the feeling of being free to make decisions based on one’s own beliefs and values,^33,34^ as well as the sense of acting without pressure or external influences.^35^ Autonomy can be targeted by engaging pharmacists with meaningful stories or allowing them to create customized avatars and profiles.^29,30,32,36^ Competence refers to the feelings of success, efficiency and a growing mastery of a certain behaviour.^37^ For pharmacists, competence can be targeted through positive feedback and quiz-type challenges.^36^ Social relatedness is the feeling of connectedness with others and the sense of belonging to a social environment.^23,28,30^ Pharmacist discussion forums and peer support networks can establish a sense of social relatedness by increasing pharmacists’ cooperation and social interaction.^29,30,32,36^ Hence, by combining key psychological drivers for human behaviour such as achievement, rewards and cooperation, gamification creates desired changes in behaviours.^26^

## Designing a successful gamified intervention for pharmacists

A gamified intervention’s success in achieving a desired behaviour depends on the game elements that are selected and incorporated into the intervention.^38^ The choice of game elements will depend on the targets and goals of the intervention. Most gamified systems focus on adding extrinsic motivation game elements such as points, badges and leaderboards to create immediate and short-term impact. However, if the goal is to achieve long-term impacts on a behaviour, deeper game elements such as engaging stories, role-play and customised feedback should be used to help users develop intrinsic motivation toward that behaviour.

Another factor that a gamified intervention’s success depends on is its perceived meaningfulness. The game’s elements and purpose should align with users’ needs and goals for it to be perceived as meaningful.^39,40^ Meaningful gamification focuses on game elements that users can relate to and that can positively change their mindset. Furthermore, with meaningful gamification, game elements invoke appreciation for the desired behaviour by allowing users to make relevant connections between the game and users’ desired goals.^41^ For example, pharmacists may not find earning points or badges to be as meaningful as charitable rewards. Customized feedback on performance may align better with pharmacists’ needs and thus be perceived as more meaningful and more related to their scope of practice. Thus, meaningful gamification for pharmacists should aim to provide a richer connection with the real world to achieve a long-lasting change in the desired behaviours.^24^

Overall, creating a gamified intervention for pharmacists is not an easy task. The bulk of existing research focuses on gamified interventions designed for younger users such as university students, leaving little information about game elements that would best influence professionals such as pharmacists. However, from the examples discussed above, it is clear that well-designed gamified interventions use game elements that target both extrinsic and intrinsic motivation. Extrinsic motivation targeted by rewards (e.g., points, badges) can be used to get users to start the desired behaviours. Rewards can then be replaced with more meaningful elements such as feedback and meaningful stories to target their intrinsic motivation and maintain their changes in behaviour. Therefore, gamification, by targeting extrinsic and intrinsic motivation, has a great potential to motivate and sustain desired professional behaviours among pharmacists. Researchers in the field of HCP development need to consider incorporating gamification into continuing professional development activities to achieve a true and sustained change. ■
